# Vision Sensor-Based Road Detection for Field Robot Navigation

**DOI:** 10.3390/s151129594

**Published:** 2015-11-24

**Authors:** Keyu Lu, Jian Li, Xiangjing An, Hangen He

**Affiliations:** College of Mechatronic Engineering and Automation, National University of Defense Technology, Changsha 410073, Hunan, China; E-Mails: lijian@nudt.edu.cn (J.L.); anxiangjing@gmail.com (X.A.); hangenhe@yahoo.com (H.H.)

**Keywords:** robot navigation, road detection, MPGA, GrowCut, conditional random field

## Abstract

Road detection is an essential component of field robot navigation systems. Vision sensors play an important role in road detection for their great potential in environmental perception. In this paper, we propose a hierarchical vision sensor-based method for robust road detection in challenging road scenes. More specifically, for a given road image captured by an on-board vision sensor, we introduce a multiple population genetic algorithm (MPGA)-based approach for efficient road vanishing point detection. Superpixel-level seeds are then selected in an unsupervised way using a clustering strategy. Then, according to the GrowCut framework, the seeds proliferate and iteratively try to occupy their neighbors. After convergence, the initial road segment is obtained. Finally, in order to achieve a globally-consistent road segment, the initial road segment is refined using the conditional random field (CRF) framework, which integrates high-level information into road detection. We perform several experiments to evaluate the common performance, scale sensitivity and noise sensitivity of the proposed method. The experimental results demonstrate that the proposed method exhibits high robustness compared to the state of the art.

## 1. Introduction

Road detection is a fundamental issue in field robot navigation systems, which have attracted keen attention in the past several decades. Vision sensors play an important role in road detection. Image data captured by vision sensors contains rich information, such as luminance, color, texture, *etc*. Thus, vision sensors have a great potentiality in road detection [1]. Moreover, vision sensors are inexpensive compared to other popular road detection sensors, such as LiDAR and millimeter-wave radar [2].

For these reasons, many state-of-the-art field robot systems employ vision sensors for road detection. For example, Xu *et al.* [3] presented a mobile robot using a vision system to navigate in an unstructured environment. The vision system consisted of two cameras; one is used for road region detection, and the other is used for road direction estimation. Rasmussen [4] introduced a vehicle-based mobile robot system, which has achieved success in the DARPA Grand Challenge. Vision sensors mounted on the top of the windshield were used to detect the road vanishing point for steering control.

Vision sensor-based road detection is a binary labeling problem trying to label every pixel in the given road image with the category (road or background) to which it belongs [5]. However, vision sensor-based road detection is still a challenging job due to the diversity of road scenes with different geometric characteristics (varying colors and textures) and imaging conditions (different illuminations, viewpoints and weather conditions) [5].

The problem of vision sensor-based road detection has been intensively studied in recent years. Some methods are based on color and texture features, e.g., the method presented in [6] uses the HSI color space as the features for road detection, while the algorithm proposed in [7] combines texture and color features. However, in many off-road environments, the texture and color features of the road and its surroundings are quite complex and diverse, and sometimes, it is extremely difficult to distinguish road regions from the surroundings by using only texture and color features. Another approach for road detection is based on road boundaries; the proposed method in [8] used road boundaries to fit a road curvature model for road detection. Nevertheless, this kind of approach does not appropriately behave when there is no evident borders (e.g., unstructured roads). More recently, the vanishing point was used for road detection in [9]. This kind of method does not work well when there is no obvious road vanishing point or the road has curved boundaries [5]. To deal with curved boundaries, in [10], the authors proposed using the illuminant invariance to detect road regions. This approach is robust to illuminations, shadows and curved roads. However, it contain less information on road shape priors and is sensitive to noise. To make sensible use of prior information, in [11], road priors obtained from geographic information systems (GISs) are combined with the road cues estimated from the current image to achieve robust road segmentation. However, the method may fail when there is no GIS database. Without GIS or a map, Sotelo *et al.* [12] used road shape restrictions to enhance the road segmentation. To make better use of road shape priors, He *et al.* [5] proposed to use road shape priors for the road segmentation by encoding the priors into a graph-cut framework, but the method would be suboptimal when the features of the road and background are similar.

**Figure 1:**
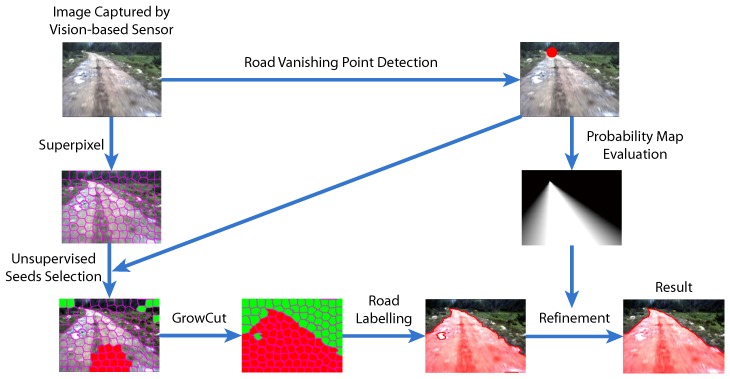
Diagram of our vision sensor-based road detection model.

In this paper, we introduce a hierarchical vision sensor-based road detection model to address this problem. More specifically, the proposed approach is depicted in Figure 1, which consists of three main components:

(1) Road vanishing point detection based on MPGA: We propose an efficient and effective road vanishing point detection method, which employed the multiple population genetic algorithm (MPGA) to search for vanishing point candidates heuristically. The value of the fitness function of MPGA is obtained by a locally-tangent-based voting scheme. In this way, we only need to estimate the local dominant texture orientations and calculate voting values at the positions of vanishing point candidate. Thus, the proposed method is highly efficient compared to traditional vanishing point detection methods. In this paper, the road vanishing point is a key element of subsequent image processing tasks.
(2) GrowCut-based road segmentation: The initial road segments are obtained using GrowCut [13], which is an interactive segmentation framework based on cellular automaton (CA) theory [14]. The seed points of GrowCut are selected automatically by using the information of the road vanishing point, which makes GrowCut become an unsupervised process without an interactive property. Seed selection and GrowCut are performed at the superpixel level. Each superpixel is regarded as a cell with a label (road or background), the initial road segment is obtained when the proliferation of cells stops.
(3) Refinement using high-level information: In order to get rid of the shortcomings of the illuminant invariance-based method [11] and to ensure that the road segments are globally consistent, inspired by [5], we employ a conditional random field (CRF) [15] to integrate some high-level information into the road segments.

The rest of this paper is organized as follows. In Section 2, Section 3 and Section 4, we introduce the proposed road detection method. Experimental results and a discussion are presented in Section 5, and conclusions are drawn in Section 6.

## 2. Road Vanishing Point Detection Based on MPGA

In a 2D image data captured by a vision sensor, the road vanishing point is the intersection of the projections of certain parallel lines (e.g., road edges, lane markings) in the real world. The road vanishing point plays an important role in road detection. The information of road direction can be obtained from the road vanishing point. Rasmussen *et al*. [16] use it for navigation directly. Moreover, the road vanishing point provides a strong clue to the localization of the road region. Hui Kong *et al*. [9] use the road vanishing point for road segmentation. In this paper, road vanishing point detection is a vital step for the whole road detection task. This section presents a robust method for the fast estimation of the road vanishing point.

### 2.1. Searching Based on MPGA

Most texture-based vanishing point detection methods [9,16,17] obtain vanishing point candidates in a greedy way, which is computationally expensive. In the proposed method, the multiple population genetic algorithm (MPGA) is employed to search vanishing point candidates heuristically. After the vanishing point candidates are obtained, we just need to estimate the local dominant texture orientations of the vanishing point candidates and their voters, after which the voting value of the vanishing point candidate can be obtained by the local dominant texture orientations.

There are some modern optimization algorithms (such as the multiple population genetic algorithm (MPGA), particle swarm algorithm optimization (PSO), the simulated annealing algorithm (SA), *etc*.) that are mainly employed for difficult optimization problem solving. The work in [18] performed experiments to evaluate the performance of some modern optimization algorithms. It concluded that these modern optimization algorithms are effective, but the genetic algorithm (GA) is more efficient than PSO and SA. The genetic algorithm [19] is widely used in optimization problems. In the algorithm, problem parameters are encoded to chromosomes, and the solution can be obtained when the evolution of the chromosomes is stopped. However, the traditional genetic algorithm may lead to local optimal solutions, and its convergence speed is slow. The multiple population genetic algorithm (MPGA) was proposed to overcome the shortcomings of the traditional genetic algorithm. Instead of starting from one population, MPGA starts from two or more populations, and each population evolves in parallel. For this reason, we apply MPGA to search for vanishing point candidates.

In order to obtain chromosomes, the coordinates of the pixels are transformed to a binary encoding. Having obtained chromosomes, we next generate *M* initial populations, and each population contains *N* chromosomes. The fitness function f(x,y) of the MPGA is defined as follows:

(1) $$f\left(x,y\right)=\sum_{i=1}^{n}Vote(P_{i},V_{j})$$

where $Vote(P_{i},V_{j})$ represents the voting value of the vanishing point candidate $V_{j}$ at (x,y), $P_{i}$ is a voter of $V_{j}$ and *n* denotes the number of voters in the voting region of $V_{j}$. The obtainment of voting values will be discussed in the next subsection.

### 2.2. Voting Scheme

After a vanishing point candidate is obtained, the local dominant texture orientation of the vanishing point candidate and its voters need to be estimated. Similar to the work in [16], Gabor filter banks are applied to estimate the local dominant texture orientation. For an orientation *ϕ* and a scale *w*, the Gabor kernels are defined by:

(2) $$\psi_{w,\phi }\left(x,y\right)=\frac{w}{\sqrt{2\pi }c}e^{-w^{2}\left(4a^{2}+b^{2}\right)/\left(8c^{2}\right)}\left(e^{iaw}-e^{-c^{2}/2}\right)$$

where a=xcosϕ+ysinϕ,b=-xsinϕ+ycosϕ. The local dominant texture orientation of each vanishing point candidate is obtained by Gabor filter banks of 36 orientations (180 divided by five). The estimation method is explained in detail in [9]. In our work, the local dominant texture orientation of a pixel is only computed once, and the value is saved for the subsequent computing.

**Figure 2:**
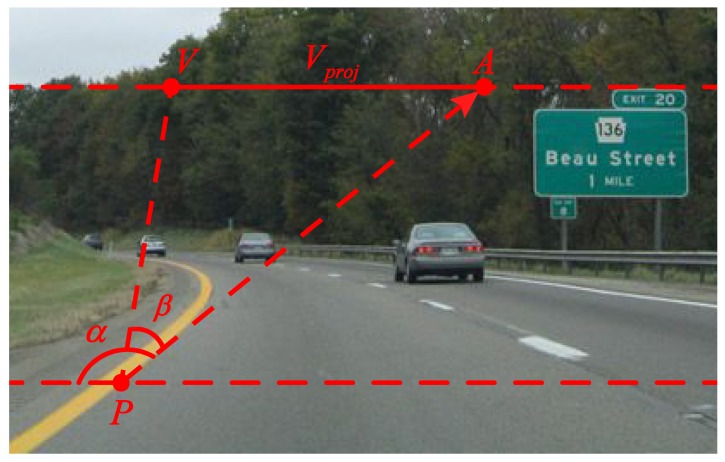
Illustration of the voting scheme.

After the local dominant texture orientation of the vanishing point candidate $V_{j}$ is obtained, its voting value $Vote(P_{i},V_{j})$ can be computed. As shown in Figure 2, *V* is a vanishing point candidate, *P* is a voter of *V* and $\vec{PA}$ is the local dominant texture orientation vector of *P*. *β* equals ∠VAP. We define $V_{proj}$ as the distance of *V* and *A*.

As shown in Figure 2, $\left(V_{x},V_{y}\right)$ and $\left(P_{x},P_{y}\right)$ are defined as the coordinates of *V* and *P*, respectively. $V_{proj}$ can be obtained as follows:

(3) $$V_{proj}=P_{x}-V_{x}+\frac{V_{y}-P_{y}}{\tan(180-\alpha )}$$

After getting the value of $V_{proj}$, our voting scheme can be defined as follows:

(4) $$Vote\left(P,V\right)=\left\{\begin{array}{cc} \; & \;\;\;\;0\;\;\;\;V_{proj}>\frac{ImageWide}{2}\\ \; & \frac{1}{1+V_{proj}^{2}}\;otherwise \end{array}\right.$$

where ImageWide represents the width of the image, *V* is a vanishing point candidate and *P* is a voter of *V*. The definition of the local voting region is similar to the soft-voting scheme in [9], which defines the voting regions as the half-disk below *V*. All of the pixels in the local voting region are used as voters.

**Figure 3:**
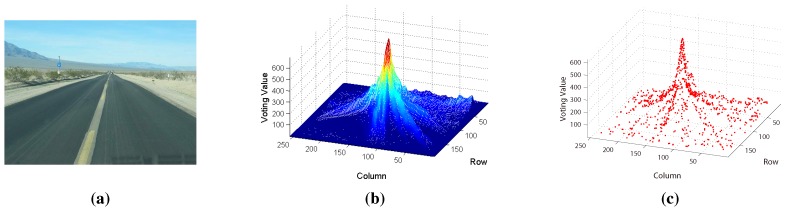
Illustration of voting: (**a**) the original road image; (**b**) the greedy voting map; (**c**) the MPGA voting map.

For better comparison, like traditional texture-based vanishing point detection methods, we compute $Vote\left(P,V\right)$ at every pixel in the test image using Equation (4), where $V_{proj}$ is obtained from Equation (3). The results are shown in Figure 3b, which illustrates that the vanishing point is the maximum value in the voting space. For MPGA-based voting, we only compute the voting value at the vanishing point candidate using Equation (4) and then search the next vanishing point candidate based on MPGA according to the result of Equation (4). Each vanishing point candidate is shown in Figure 3c (marked with red color). From Figure 3, we can see that every population experiences the processes of selection, reproduction and mutation by the fitness function; the maximum value could be found at last, and the road vanishing point is estimated at the location of the maximum value.

As we just need to estimate the local dominant texture orientations and compute voting values at the positions of the vanishing point candidate, the proposed method is more efficient than traditional texture-based methods. For a quantitative comparison, let MP and NI denote the number of initial populations and the number of chromosomes in each population, respectively. The number of vanishing point candidates is denoted by NV. For the road images of size 320×240, the NV of traditional texture-based methods is about:

(5) NV=320×240=76,800

We perform an experiment to validate the efficiency of our MPGA-based voting strategy. As shown in Table 1, NV of our voting strategy is much less than that of traditional texture-based methods (76,800). This means that our voting strategy spends less time on voting. Thus, the proposed method is more efficient than the traditional one.

**Table 1:** The result of efficiency validation.

		NI	10	20	30	40	50
	NV						
MP							
10			418	1009	1553	2139	2674
20			731	1767	2716	3738	4594
30			1004	2439	3761	5132	6313
40			1290	3098	4717	6427	7797
50			1537	3681	5597	7571	9177

**Figure 4:**
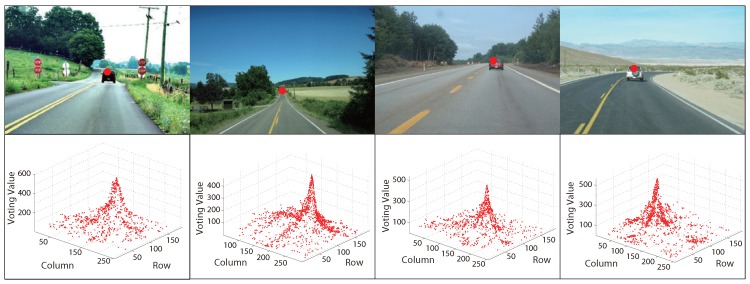
Examples of MPGA-based vanishing point detection in structured roads.

**Figure 5:**
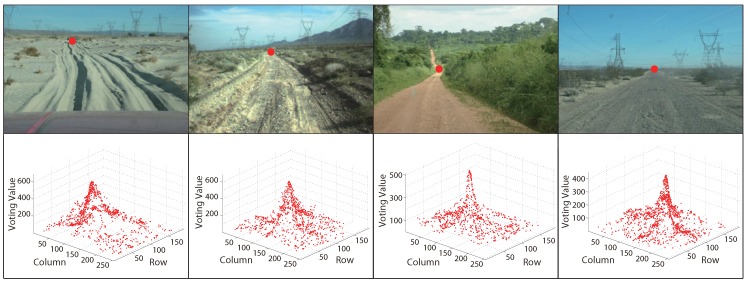
Examples of MPGA-based vanishing point detection in unstructured roads.

Figure 4 and Figure 5 are some examples of MPGA vanishing point detection in structured roads and unstructured roads, respectively. The red points in the first rows of the figures are the obtained road vanishing point. The second rows are the corresponding MPGA voting maps.

## 3. GrowCut-Based Road Segmentation

The initial road segments are obtained using GrowCut [13], which is an interactive segmentation framework based on cellular automaton (CA) theory [14]. GrowCut starts with a set of seed points, and the seed points iteratively try to occupy their neighbors according to cellular automaton, until convergence. Because of its robustness, GrowCut is widely used in image segmentation [13], object detection [20], *etc*. In this paper, the seed points of GrowCut are selected automatically by using the information of the road vanishing point, which makes GrowCut become an unsupervised process without an interactive property.

### 3.1. Seed Selection at the Superpixel Level

**Figure 6:**
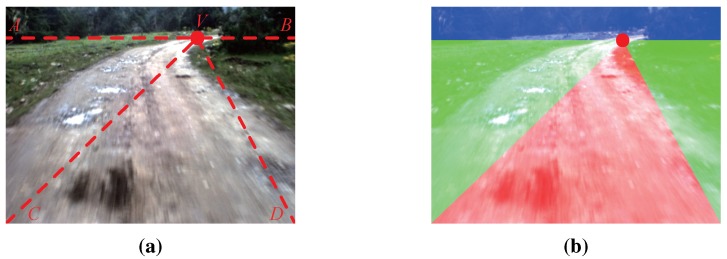
Clustering region: (**a**) illustration of the clustering region definition; (**b**) example of the clustering region definition.

Seeds are the starting points of GrowCut. In our work, the seeds are selected automatically by using the K-means clustering algorithm [21]. The road and the background clustering region are defined by using the information of the road vanishing point, which can be obtained efficiently by the method introduced in Section 2. As shown in Figure 6a, *V* denotes the road vanishing point of the given image and AB is the horizontal line crossing the road vanishing point *V* (called the vanishing line). Let *C* and *D* denote the bottom left corner and the bottom right corner of the given image, respectively. The lines between *V*, *C* and *D* divide the area below AB into three regions: VAC, VCD and VBD.

For images captured by on-board cameras, most pixels in the region VCD belong to the road surface, and most pixels in the region VAC and VBD belong to the background. The region VCD is defined as the road clustering region, while VAC and VBD are the background clustering region. As shown in Figure 6b, the red area is the road clustering region; the green area is the background clustering region; and the blue area is the sky region (the region above vanishing line AB).

**Figure 7:**
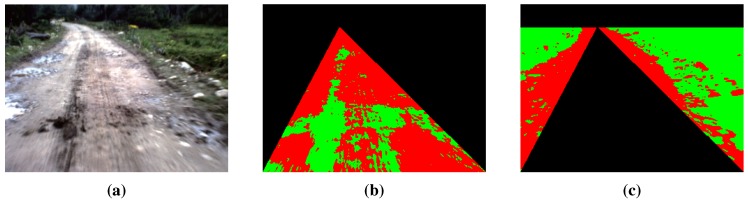
Road and background region clustering: (**a**) test road image; (**b**) road region clustering; (**c**) background region clustering.

We use three channels (R, G and B) of the given color road image as the features of clustering. After the clustering region is defined, the K-means clustering algorithm [21] is applied to the road and the background clustering regions, respectively (the initial points of clustering are selected randomly). Road and background region clustering is illustrated in Figure 7, where Figure 7a is the original road image and Figure 7b,c are the results of RGB feature-based K-means clustering. For the result of road region clustering in Figure 7b, the proportion of the red category is larger than that of the green category. This means that in the result of road region clustering, the category with a larger proportion can be seen as the representative of the road surface. According to this assumption, road seeds of GrowCut are selected from the category with a larger proportion in the result of the road region clustering (the red category in Figure 7b). Similarly, background seeds are selected from the category with a larger proportion in the result of the background region clustering (the green category in Figure 7c).

In order to produce spatially more appealing road segments, inspired by [22], we select seeds and apply GrowCut at the superpixel level. We perform a preliminary over-segmentation of the given road image into superpixels using the SLICalgorithm [23]. Rather than using the pixel grids and rectangular patches, superpixels are likely to be uniform in color and texture and tend to preserve boundaries, so they are more perceptually meaningful and representationally efficient [24]. Furthermore, using superpixels may dramatically reduce the computational complexity of subsequent image processing tasks (such as GrowCut) [22].

In the given road image, for the *k*-th superpixel $S_{k}$, $Cr_{k}$ denotes the ratio of the number of pixels belonging to road seeds and the total number of pixels in this superpixel and takes the form:

(6) $$Cr_{k}=\frac{\sum_{i\in S_{k}}l_{i}}{N_{k}}$$

where $l_{i}\in \left\{0\left(background\right),1\left(road\right)\right\}$ denotes the label of the *i*-th pixel and $N_{k}$ is the total number of pixels in $S_{k}$.

Let $\left(x_{k},y_{k}\right)$ denote the mean coordinates of the pixels in $S_{k}$ and $\left(x_{m},y_{m}\right)$ be the coordinates of the pixel in the bottom center of the given road image. For the selection of road seeds, the normalized distance between $S_{k}$ and $\left(x_{m},y_{m}\right)$ is denoted by $Dr_{k}$, which can be defined as follows:

(7) $$Dr_{k}=\frac{\sqrt{{\left(x_{m}-x_{k}\right)}^{2}+{\left(y_{m}-y_{k}\right)}^{2}}}{\sqrt{x_{m}^{2}+y_{m}^{2}}}$$

Let $Pr_{k}$ be the probability of $S_{k}$ being selected as a road seed:

(8) $$Pr_{k}=\frac{Cr_{k}+\left(1-Dr_{k}\right)\cdot Tr}{1+Tr}$$

where Tr is the factor controlling the weight of $\left(1-Dr_{k}\right)$ in computing $Pr_{k}$ (Tr=0.01 in this work). Due to the fact that superpixels closer to the bottom center of the on-board road image have a higher probability of being road seeds, $Dr_{k}$ should be taken into consideration in computing the probability of being road seeds.

**Figure 8:**
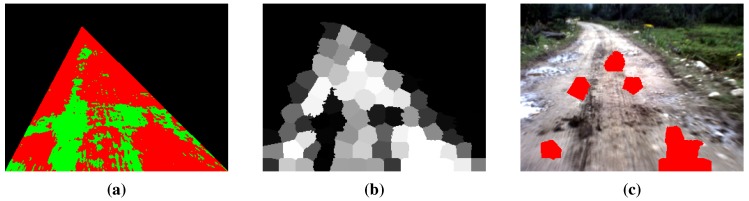
Illustration of superpixel-level road seed selection: (**a**) the result of road region clustering; (**b**) the probability of being road seeds (the brighter the superpixel, the higher the probability); (**c**) the result of superpixel-level road seed selection.

For the result of the road region clustering in Figure 8a, the probability of each superpixel being selected as a road seed can be obtained by Equation (8) (see Figure 8b, which is called the probability map). The result of superpixel-level road seed selection is illustrated in Figure 8c.

**Figure 9:**
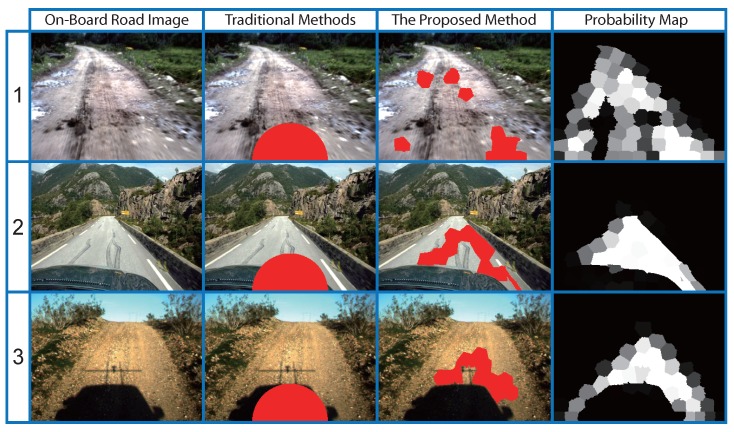
Comparisons with the traditional seed selection method.

Compared to most traditional seed selection methods, the proposed method is highly robust. With the common assumption [25], traditional methods define a “safe” window in the road image and assume that the pixels in the “safe” window belong to the road pattern (see Figure 9; the “safe” window is a semi-circular region at the center-bottom of the on-board road image). As shown in Figure 9, traditional methods may not work well when the features of the road and its surroundings are complex and diverse, where they are likely to select seeds in some incorrect regions, such as shadow and some parts of the car.

Similarly, for background seeds, let $Cg_{k}$ be the ratio of the number of pixels belonging to background seeds and the total number of pixels in the superpixel $S_{k}$:

(9) $$Cg_{k}=\frac{\sum_{i\in S_{k}}|l_{i}-1|}{N_{k}}$$

In order to make background seeds be equally distributed on the left and right side of the road image, the background clustering region is divided into two parts, denoted by Bl and Br, which represent the parts on the left and right side of the road clustering region, respectively. Let $\left(x_{l},y_{l}\right)$ and $\left(x_{r},y_{r}\right)$ be the coordinates in the top left corner and top right corner of the background clustering region, respectively. $\left(x_{\max},y_{\max}\right)$ denote the coordinates in the bottom right corner of the given road image. For the selection of background seeds, the normalized distance $Dg_{k}$ takes the form:

(10) $$Dg_{k}=\left\{\begin{array}{cc} \; & \frac{\sqrt{{\left(x_{l}-x_{k}\right)}^{2}+{\left(y_{l}-y_{k}\right)}^{2}}}{\sqrt{x_{\max^{2}}+y_{\max^{2}}}}\;\;k\in Bl\\ \; & \frac{\sqrt{{\left(x_{r}-x_{k}\right)}^{2}+{\left(y_{r}-y_{k}\right)}^{2}}}{\sqrt{x_{\max^{2}}+y_{\max^{2}}}}\;k\in Br \end{array}\right.$$

The probability of $S_{k}$ being selected as a background seed can be defined as follows:

(11) $$Pg_{k}=\frac{Cg_{k}+\left(1-Dg_{k}\right)\cdot Tg}{1+Tg}$$

where Tg is the factor controlling the weight of $\left(1-Dg_{k}\right)$ in computing $Pg_{k}$ (Tg=0.01 in this work).

Clustering-based background seed selection aims at selecting background seeds outside the road regions. Although clustering regions have many kinds of textures, background seeds are only selected from the category with a larger proportion in the result of the background region clustering, which is less likely to belong to the road pattern. Besides, Equations (10) and (11) penalize the superpixels close to road regions. In these ways, clustering-based background seed selection is able to work well.

**Figure 10:**
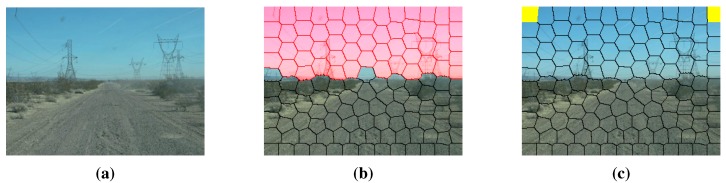
The selection of superpixel-level background seeds above a vanishing line: (**a**) test road image; (**b**) superpixels above the vanishing line (colored in red); (**c**) the result of the selection (colored in yellow).

**Figure 11:**
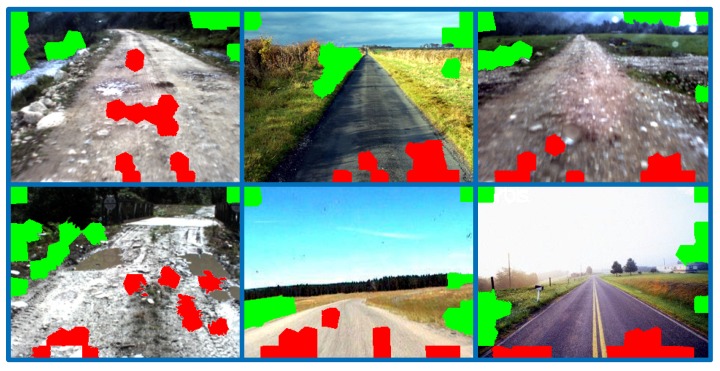
The results of superpixel-level seed selection (road seeds are colored in red; background seeds are colored in green).

For on-board road images, according to the fact that the sky is likely to appear in the region above the vanishing line (the blue area in Figure 6b) and that the features of this region are quite different from that of the road and background clustering region, we need to select two background seeds in the region above the vanishing line. Let $S_{sky1}$ and $S_{sky2}$ be two superpixel-level background seeds, which are selected by Equation (12).

(12) $$\left\{\begin{array}{cc} sky1 & =\underset{k}{\arg\min}\sqrt{{\left(x_{k}-1\right)}^{2}+{\left(y_{k}-1\right)}^{2}}\\ sky2 & =\underset{k}{\arg\min}\sqrt{{\left(x_{k}-x_{\max}\right)}^{2}+{\left(y_{k}-1\right)}^{2}} \end{array}\right.$$

where (1,1) and $\left(x_{\max},1\right)$ are the coordinates in the top left corner and top right corner of the road images. The selection of $S_{sky1}$ and $S_{sky2}$ is illustrated in Figure 10, and some qualitative results of seed selection at the superpixel level are illustrated in Figure 11.

### 3.2. Segmentation Using the GrowCut Framework

After seed points are obtained, the seed superpixels iteratively try to occupy their neighbors according to GrowCut [14] at the superpixel level until convergence. For the superpixel $S_{i}$, let $Sl_{i}$ be its label (road or background), where $Sl_{i}\in \left\{-1\left(background\right),0\left(undetermined\right),1\left(road\right)\right\}$. $\vec{F_{i}}$ denotes the feature vector of the superpixel $S_{i}$ and $\theta_{i}$ denotes the strength ($\theta_{i}\in \left[0,1\right]$), which stands for the ability to attack or defend. Thus, the state of the superpixel $S_{i}$ can be defined by a triplet $\left(Sl_{i},\theta_{i},\vec{F_{i}}\right)$. Initially, the label of each superpixel is set as:

(13) $$Sl_{i}=\left\{\begin{array}{cc} 1 & \;\;\;S_{i}\in Sr\\ -1 & \;\;\;S_{i}\in Sg\\ 0 & \;\;\;S_{i}\in \bar{Sr\cup Sg} \end{array}\right.$$

where Sr and Sg denote the sets of road and background seeds, respectively. the initial value of strength is set as:

(14) $$\theta_{i}=\left\{\begin{array}{cc} \; & 1\;S_{i}\in Sr\cup Sg\\ \; & 0\;S_{i}\in \bar{Sr\cup Sg} \end{array}\right.$$

Moreover, we need to define the distance between two feature vectors. Álvarez *et al*. had demonstrated that intraclass variability caused by lighting conditions is a major challenge for road detection [10]. As a field robot may work under different lighting conditions (e.g., different illuminations, weather and shadow conditions), in this work, illuminant invariance features [10] and RGB color features are used to describe each superpixel. Illuminant invariance features are able to make the road detection more robust in shadowy and varying illumination conditions. For each pixel in a color image, illuminant invariance feature $I_{i}$ can be obtained by:

(15) $$\left\{\begin{array}{cc} I_{i} & =e^{\beta }\\ \beta & =\log\left(\frac{R_{i}}{G_{i}+1}\right)\cdot \cos\left(\theta \right)+\log\left(\frac{B_{i}}{G_{i}+1}\right)\cdot \sin\left(\theta \right) \end{array}\right.$$

where $\left(R_{i},G_{i},B_{i}\right)$ denotes the RGB color feature of the *i*-th pixel. The illuminant invariance feature of the *i*-th superpixel is defined as follows:

(16) $$FI_{i}=\frac{\sum_{k\in S_{i}}^{n}I_{k}}{n_{i}}$$

where $n_{i}$ denotes the number of pixels in superpixel $S_{i}$. Similarly, the RGB color feature of the *i*-th superpixel can be obtained by:

(17) $$FR_{i}=\frac{\sum_{k\in S_{i}}^{n}R_{k}}{n_{i}},FG_{i}=\frac{\sum_{k\in S_{i}}^{n}G_{k}}{n_{i}},FB_{i}=\frac{\sum_{k\in S_{i}}^{n}B_{k}}{n_{i}}$$

The distance between feature vectors *i* and *j* is denoted by $Dm_{i,j}$ and takes the form:

(18) $$Dm_{i,j}=\frac{|FI_{i}-FI_{j}|+Km\cdot \sqrt{{\left(FR_{i}-FR_{j}\right)}^{2}+{\left(FG_{i}-FG_{j}\right)}^{2}+{\left(FB_{i}-FB_{j}\right)}^{2}}}{1+Km}$$

where Km is the factor controlling the weight of the RGB color feature in computing $Dm_{i,j}$ (Km=0.2 in this work).

According to the GrowCut [14] framework, each superpixel is regarded as a cell, and cells with labels 1 and -1 proliferate from seeds. In the process of proliferation, they attack and occupy their differently-labeled neighbors. Let $S_{i}$ and $S_{j}$ be two neighbor cells with different labels ($\left(Sl_{i}\neq Sl_{j}\right)\wedge \left(Sl_{i}\neq 0\right)\wedge \left(Sl_{j}\neq 0\right)$). Let $S_{i}$ be the attacking cell and $S_{j}$ the attacked cell; then, $S_{i}$ occupies $S_{j}$ if:

(19) $$g\left(Dm_{i,j}\right)\cdot \theta_{i}>\theta_{j}$$

where g(x) is a monotonically-decreasing function:

(20) $$g\left(x\right)=1-\frac{x}{\max{∥Dm_{i,j}∥}_{2}}$$

If $S_{i}$ occupies $S_{j}$, then the label and strength of $S_{j}$ will change according to the state of $S_{i}$:

(21) $$\left\{\begin{array}{cc} \; & Sl_{j}=Sl_{i}\\ \; & \theta_{j}=g\left(Dm_{i,j}\right)\cdot \theta_{i} \end{array}\right.$$

**Figure 12:**
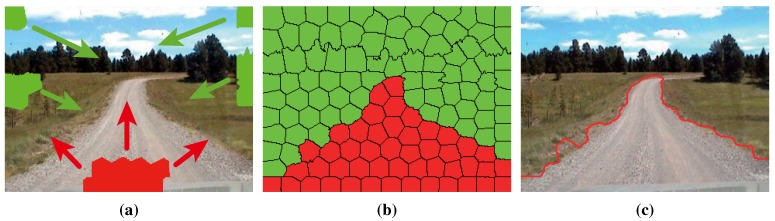
Illustration of segmentation using GrowCut: (**a**) the proliferation of seeds; (**b**) the convergence of the iteration; (**c**) the contour of the obtained road segments.

Each superpixel in the given road image tries to occupy its differently-labeled neighbors iteratively until all of the superpixels converge to a stable configuration. As shown in Figure 12, the road segment is obtained after convergence.

## 4. Refinement Using High-Level Information

**Figure 13:**
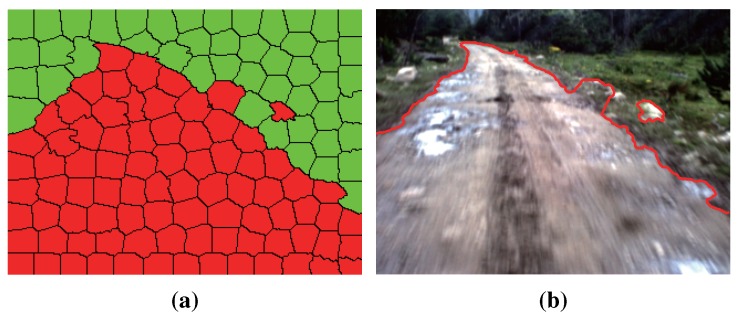
Problems with initial road segments obtained by GrowCut: (**a**) the convergence of the iteration; (**b**) the contour of road segments.

Due to the complexity of the road scene, there still may exist some isolated superpixels or unsmooth edges in the road segments obtained by GrowCut (see Figure 13). The initial road segments still need to be refined by some high-level information. Olga Veksler [26] used the information called the “star shape prior” for image segmentation. Similarly, He *et al*. [5] proposed to use the information of road shape priors for the road segmentation by encoding the information into a graph-cut framework.

Inspired by these previous works, we implement the conditional random field (CRF) [15] at the pixel level to integrate some high-level information (e.g., shape prior, road vanishing point) to achieve a more robust detection.

In this work, the high-level information integrated in the refinement is listed as follows:

- There exist no isolated area in the road segments nor background segments;
- In on-board road images, road segments are shrinking from bottom to top;
- The direction of the road is relevant to the position of the road vanishing point.

To build the CRF model at the pixel level, let $l_{i}$ denote the label (road or background) of the pixel *i* and *l* denote the set of all label assignments. The CRF energy to minimize can be written as:

(22) $$E\left(l\right)=\sum_{i\in V}\phi_{i}\left({\hat{l}}_{i},l_{i}\right)+\lambda \sum_{d_{ij}\in D}\psi_{ij}\left(l_{i},l_{j}\right)+\sum_{d_{ij}\in D}\varphi (l_{i},l_{j})+w\sum_{i\in V}\eta_{i}$$

where $\phi_{i}\left({\hat{l}}_{i},l_{i}\right)$ is the unary term enforcing the label $l_{i}$ (road or background) to take a value close to the label ${\hat{l}}_{i}$ obtained by GrowCut:

(23) $$\phi_{i}\left({\hat{l}}_{i},l_{i}\right)=\left\{\begin{array}{c} 1\;{\hat{l}}_{i}\neq l_{i}\\ 0\;{\hat{l}}_{i}=l_{i} \end{array}\right.$$

In Equation (22), $\psi_{ij}$ is the pairwise term penalizing the different assignments for neighboring pixels *i* and *j*. Let $\vec{C_{i}}$ be the RGB color feature vector of the *i*-th pixel; $\psi_{ij}$ takes the form:

(24) $$\psi_{i}\left({\hat{l}}_{i},l_{i}\right)=\left\{\begin{array}{cc} e^{-\beta {∥\vec{C_{i}}-\vec{C_{j}}∥}_{2}} & l_{i}\neq l_{j}\\ 0 & l_{i}=l_{j} \end{array}\right.$$

**Figure 14:**
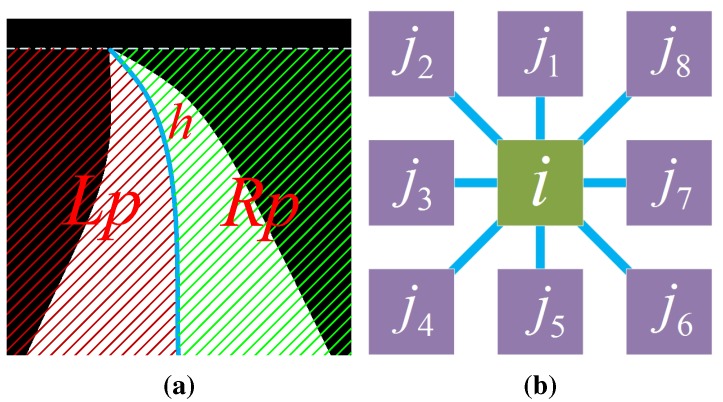
Illustration of the road shape prior: (**a**) a road segment with the middle line *h*; Lp denotes the left part of *h*; Rp denotes the right part of *h*; (**b**) a standard eight-neighborhood system with a center pixel *i* and its neighbors $j_{1}\ldots j_{8}$.

Inspired by [5], a second-order term $\varphi_{ij}$ is employed to incorporate the road shape prior into the CRF framework. To describe the road shape prior, let *h* be the middle line of the road segment obtained by GrowCut (see Figure 14a). Lp and Rp denote the left and right part of *h*, respectively. *i* is an arbitrary pixel in the road segment. $j_{1}\ldots j_{8}$ are neighbors of *i* (see Figure 14b). From the high-level information, the road shape implies that:

(25) $$\left\{\begin{array}{c} \forall i,\left(i\in Lp\right)\wedge \left(j_{3}\in Road\right)\Rightarrow i\in Road\\ \forall i,\left(j_{7}\in Lp\right)\wedge \left(i\in Road\right)\Rightarrow j_{7}\in Road\\ \forall i,\left(i\in Rp\right)\wedge \left(j_{7}\in Road\right)\Rightarrow i\in Road\\ \forall i,\left(j_{3}\in Rp\right)\wedge \left(i\in Road\right)\Rightarrow j_{3}\in Road \end{array}\right.$$

From Equation (25), the second-order term $\varphi_{ij}$ can be defined as:

(26) $$\begin{array}{c} \varphi_{ij}\left(l_{i},l_{j}\right)=\left\{\begin{array}{c} \infty \;\;\;\;\;\;\;if\;\{\left(l_{i}=0\;\;and\;\;l_{j}=1\;\;and\;\;i\in Lp\;\;and\;\;j=j_{3}\right)\\ \;\;\;\;\;\;\;\;\;\;\;\;\;\;\;or\;\;(l_{i}=1\;\;and\;\;l_{j}=0\;\;and\;\;j\in Lp\;\;and\;\;j=j_{7})\\ \;\;\;\;\;\;\;\;\;\;\;\;\;\;\;or\;\;(l_{i}=0\;\;and\;\;l_{j}=1\;\;and\;\;i\in Rp\;\;and\;\;j=j_{7})\\ \;\;\;\;\;\;\;\;\;\;\;\;\;\;\;or\;\;(l_{i}=1\;\;and\;\;l_{j}=0\;\;and\;\;j\in Rp\;\;and\;\;j=j_{3})\}\\ 0\;\;\;\;\;\;\;\;\;\;\mathrm{otherwise} \end{array}\right. \end{array}$$

**Figure 15:**
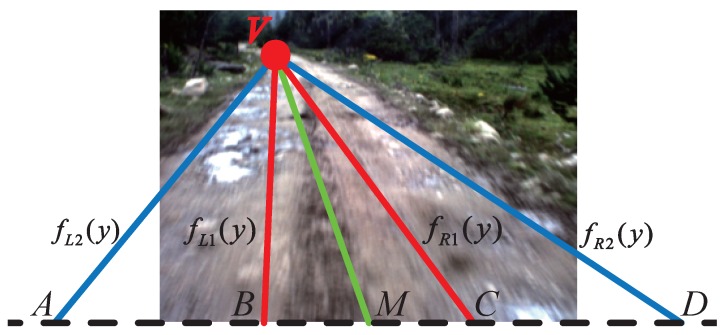
Image division for probability map calculation.

In the CRF energy Equation (22), the last term $\eta_{i}$ is used to incorporate road vanishing point information. For the calculation of $\eta_{i}$, we first need to obtain a probability map according to the position of the road vanishing point. Let $D_{p}$ and *M* respectively denote the length and the center of the projection of the road segment onto the last line of the road image. *V* denotes the road vanishing point. The road image is divided into several regions illustrated in Figure 15, where the length of BM and CM is one fourth of that of $D_{p}$, and the length of AM and DM is three fourths of that of $D_{p}$. We define the probability of the road pattern to be presented at each pixel in the region VBC is 100%. In VAB and VCD, pixels closer to the center line VM have a higher probability of being road pixels. Let $\left(x_{v},y_{v}\right)$ be the coordinates of the road vanishing point; ImH and ImW denote the height and width of the road image, respectively; $\left(x_{m},ImW\right)$ denotes the coordinates of *M*. As shown in Figure 15, $f_{L1}\left(y\right)$, $f_{L2}\left(y\right)$, $f_{R1}\left(y\right)$ and $f_{R2}\left(y\right)$ are respectively the linear functions of VB, VA, VC and VD. The linear functions takes the form:

(27) $$\left\{\begin{array}{cc} f_{L1}\left(y\right) & =\left(y-y_{v}\right)\cdot \frac{x_{v}-\left(x_{m}-0.5D_{p}\right)}{y_{v}-ImH}+x_{v}\\ f_{L2}\left(y\right) & =\left(y-y_{v}\right)\cdot \frac{x_{v}-\left(x_{m}-0.75D_{p}\right)}{y_{v}-ImH}+x_{v}\\ f_{R1}\left(y\right) & =\left(y-y_{v}\right)\cdot \frac{x_{v}-\left(x_{m}+0.5D_{p}\right)}{y_{v}-ImH}+x_{v}\\ f_{R2}\left(y\right) & =\left(y-y_{v}\right)\cdot \frac{x_{v}-\left(x_{m}+0.75D_{p}\right)}{y_{v}-ImH}+x_{v} \end{array}\right.$$

**Figure 16:**
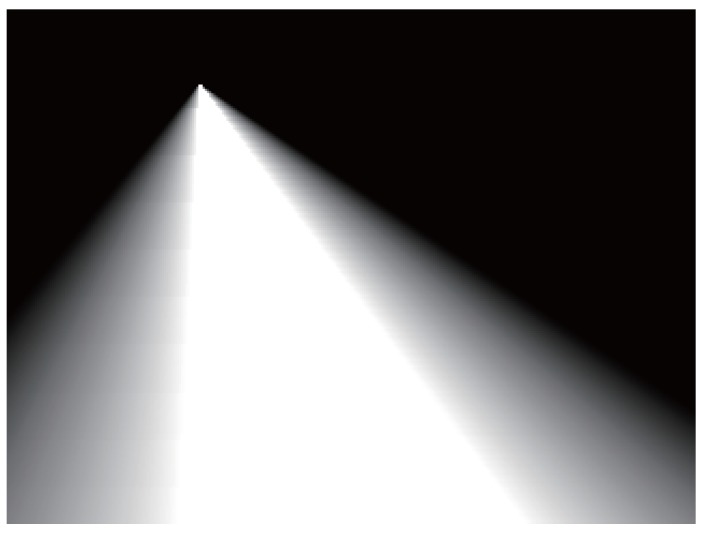
Probability map obtained according to the position of the road vanishing point (the brighter pixel stands for the higher probability).

Let $\left(x_{i},y_{i}\right)$ be the coordinates of the *i*-th pixel in the image; the last term $\eta_{i}$ of the CRF energy stands for the probability obtained according to the position of the road vanishing point and can be obtained by Equation (28). The probability map is illustrated in Figure 16.

(28) $$\eta_{i}=\left\{\begin{array}{cc} 0 & x_{i}<f_{L2}\left(y_{i}\right)\\ \frac{x_{i}-f_{L2}\left(y_{i}\right)}{f_{L1}\left(y_{i}\right)-f_{L2}\left(y_{i}\right)} & f_{L2}\left(y_{i}\right)\leq x_{i}<f_{L1}\left(y_{i}\right)\\ 1 & f_{L1}\left(y_{i}\right)\leq x_{i}\leq f_{R1}\left(y_{i}\right)\\ \frac{x_{i}-f_{R2}\left(y_{i}\right)}{f_{R1}\left(y_{i}\right)-f_{R2}\left(y_{i}\right)} & f_{R1}\left(y_{i}\right)<x_{i}\leq f_{R2}\left(y_{i}\right)\\ 0 & x_{i}>f_{R2}\left(y_{i}\right) \end{array}\right.$$

**Figure 17:**
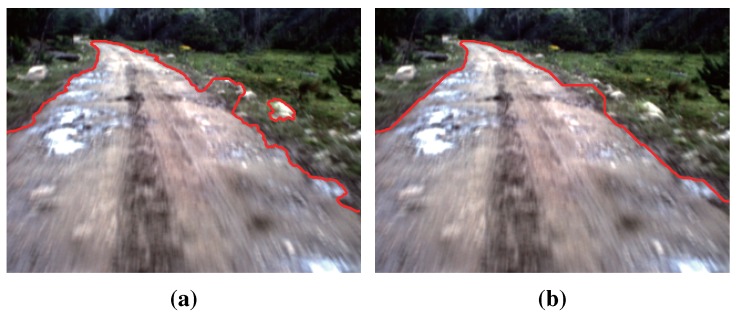
Comparison between the results of GrowCut and refinement: (**a**) the result of GrowCut; (**b**) the result of refinement.

The CRF energy E(l) in Equation (22) is minimized using Graph Cut [27]. The result of refinement is illustrated in Figure 17, which manifests that the refinement using high-level information is effective in road detection.

## 5. Results and Discussion

### 5.1. Common Performance

**Figure 18:**
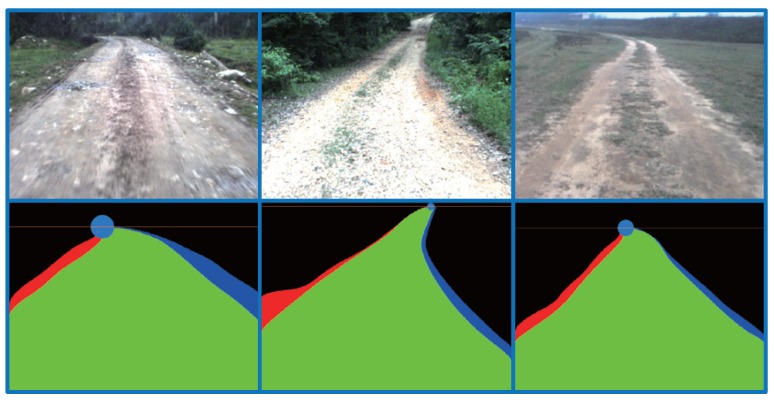
Images with ground truths in the database. The first rows are original images; the second rows are the corresponding ground truths; the road region is marked with the green color; ambiguous regions of the left and right edges are marked with red and blue colors, respectively.

To validate the proposed road detection model, we evaluate the performance of the proposed approach on the OffRoadScene database [28], which consists of 770 unstructured road images, which are captured by driving on several challenging unstructured roads. The database contains various types of road scenes with different texture, shadows, illuminations and weather conditions. All of the images are resized to 320×240 for testing. The ground truth of the database is manually labeled. As most of the unstructured roads have no clear borders, the database uses ambiguous regions to describe the road borders; as shown in Figure 18, the ambiguous regions are unconcerned with respect to the experimental results.

As shown in Table 2, quantitative evaluations are provided using four types of pixel-wise measures: precision (P), accuracy (A), false positive rate (FPR) and recall (R). In Table 2, TP and TN denote the number of road pixels correctly detected and the background pixels correctly detected, respectively. FP and FN denote the number of background pixels incorrectly marked and the road pixels incorrectly identified, respectively.

**Table 2:** Performance metric.

Pixel-Wise Measure	Definition
Precision	$P=\frac{TP}{TP+FP}$
Accuracy	$A=\frac{TP+TN}{TP+FN+FP+TN}$
False Positive Rate	$FPR=\frac{FP}{TN+FP}$
Recall	$R=\frac{TP}{TP+FN}$

In the experiment, we compare our proposed method to two state-of-the-art road detection algorithms: Kong *et al*.’s method [9] and Álvarez *et al*.’s method [10]. Quantitative results are shown in Table 3.

**Table 3:** Quantitative results on the OffRoadScene database.

Method	P(%)	A(%)	FPR(%)	R(%)
Kong *et al*.’s method [9]	98.1278	88.0466	2.1710	80.7174
Álvarez *et al*.’s method [10]	96.7897	84.9126	3.7357	76.4284
Our proposed method	**98.4708**	**97.0281**	**2.1243**	**96.5481**

### 5.2. Scale Sensitivity

**Figure 19:**
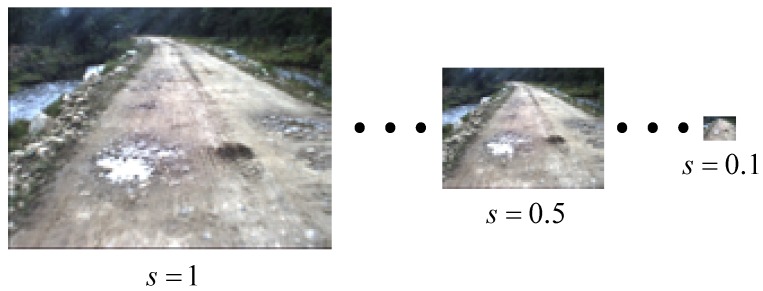
Illustration of the scale sensitivity evaluation.

In order to validate the sensitivity of algorithms to image scale, test images from the OffRoadScene database are resized to different scales. Let m×n be the size of images in the database; *s* denotes the scale factor, which resizes images to (m·s)×(n·s) (s∈[0,1]). A Gaussian filter is applied to smooth the image before resizing to a smaller scale. In the experiment, as shown in Figure 19, we resize images to 10 scales by setting *s* from 0.1 to 1 using a step-size of 0.1.

**Figure 20:**
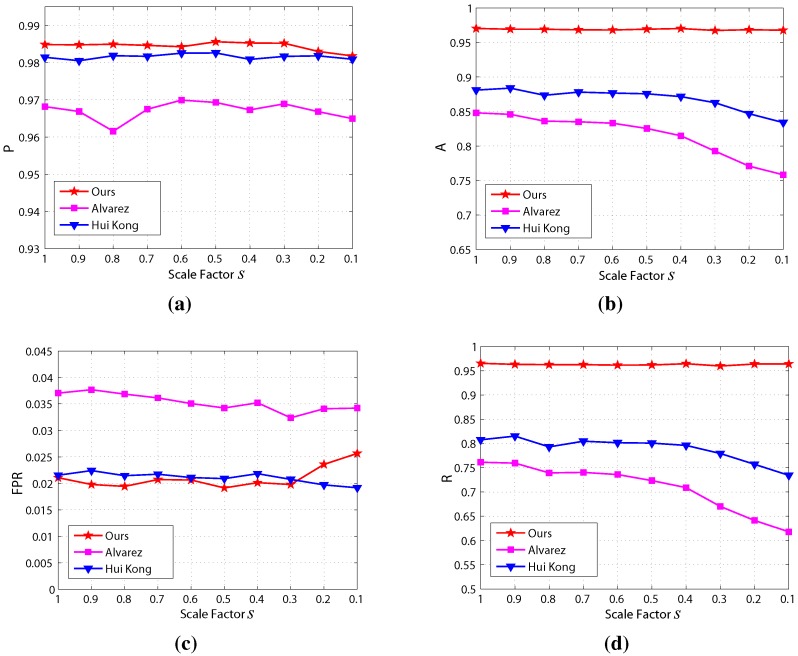
Results of the scale sensitivity evaluation: (**a**) precision; (**b**) accuracy; (**c**) false positive rate; (**d**) recall.

The results of the scale sensitivity evaluation are shown in Figure 20, which demonstrates that the precision, accuracy and recall of the proposed method are highest and and false positive rate is lowest among the three methods. Moreover, the precision, accuracy, recall and false positive rate of the proposed method are highly stable compared to the other two methods. This means that our proposed method is less sensitive to image scale.

### 5.3. Noise Sensitivity

**Figure 21:**
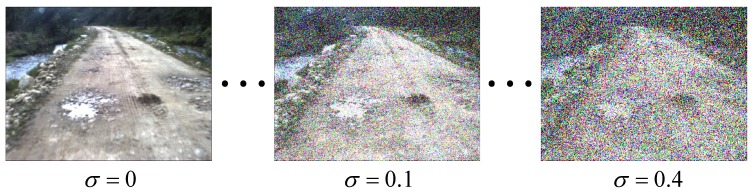
Illustration of the noise sensitivity evaluation.

There may exist noise in the images captured by the on-board camera in a real scene, which is a challenge of vision-based road detection. In this work, Gaussian noise is added to images to evaluate the noise sensitivities of the algorithms:

(29) $$\hat{I}\left(i,j,k\right)=I\left(i,j,k\right)+n\left(i,j,k\right)$$

where $\hat{I}\left(i,j,k\right)$ denotes that the noise with value n(i,j,k) is added to the *k*-th channel of the pixel located in row *i* and column *j* of the image. n(i,j,k) takes the form:

(30) $$p\left(x\right)=\frac{1}{\sigma \sqrt{2\pi }}e^{-\frac{x^{2}}{2\sigma^{2}}}$$

where *σ* denotes variance. As shown in Figure 21, we add noise with variance *σ* ranging from 0.05 to 0.4 to validate the algorithms.

**Figure 22:**
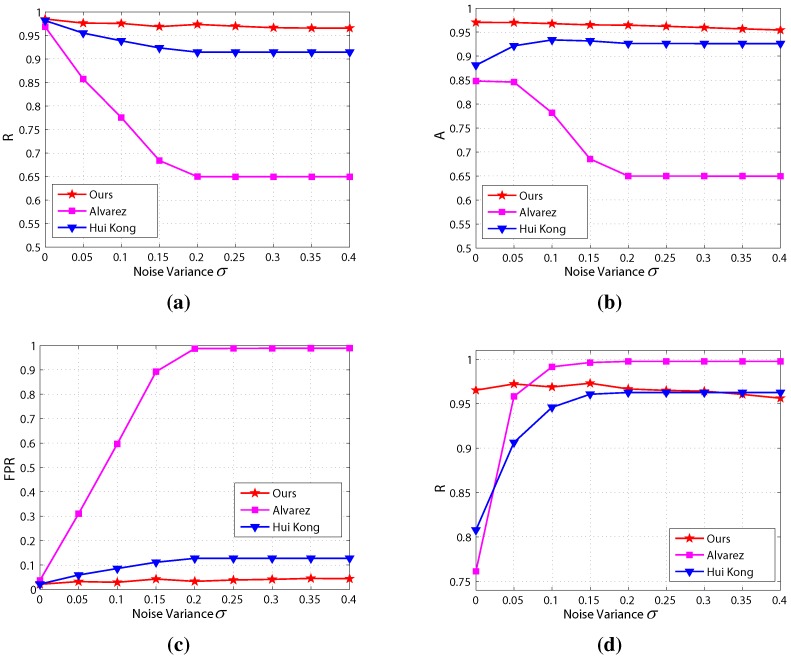
Results of the noise sensitivity evaluation: (**a**) precision; (**b**) accuracy; (**c**) false positive rate; (**d**) recall.

The results of the noise sensitivity evaluation are shown in Figure 22, which shows that the precision and accuracy of the proposed method are highest and the false positive rate is lowest among the three methods in most noise conditions. Besides, the precision, accuracy, recall and false positive rate of the proposed method are highly robust compared to the other two methods, which implies that the proposed method is less sensitive to noise.

### 5.4. Discussion

**Figure 23:**
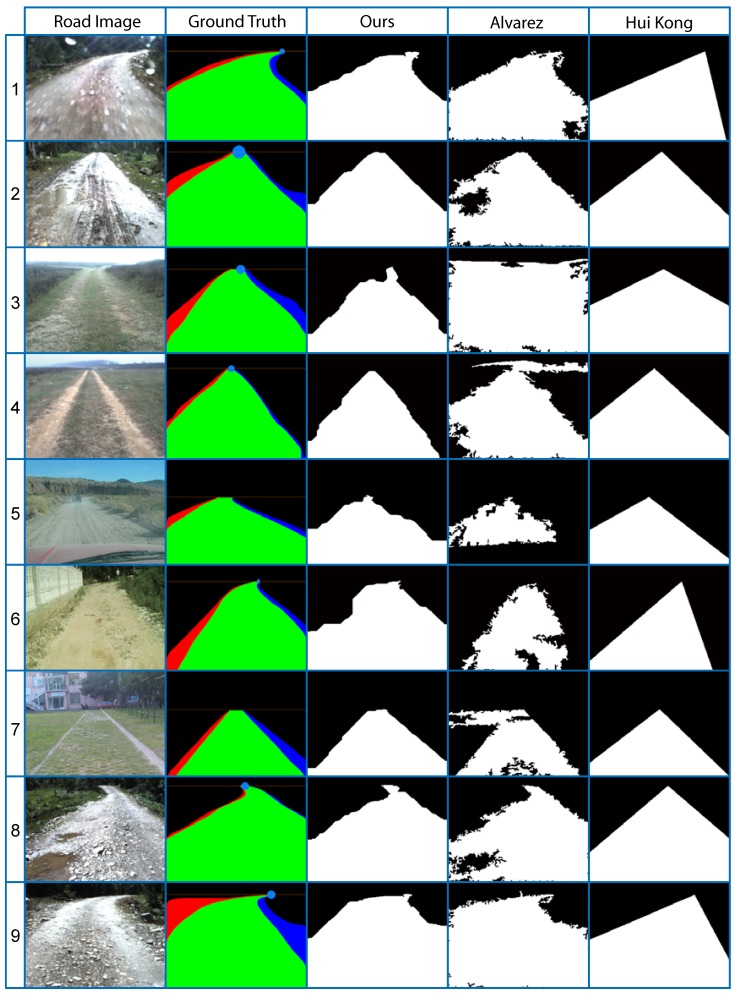
Qualitative comparisons between different methods. Road and background regions in Columns 3–5 are marked with white and black, respectively.

As shown in Table 2, Figure 20 and Figure 22, our proposed method performs much better than other methods. Some qualitative results can be seen in Figure 23, where Kong *et al*.’s method [9] is based on the road vanishing point. From the experiments, we can see that the precision of Kong *et al*.’s method is relatively high, and the false positive rate is relatively low. Besides, the method is less sensitive to scale and noise. This is due to the fact that texture-based vanishing point detection mainly depends on the edges of the main lanes instead of the details in road images, and the changes of scale and noise mainly affect the details of road images, while the edges of the main lanes are kept. However, the method may not fit well when the road boundaries are curved (see Figure 23, Rows 1 and 8).

Álvarez *et al*.’s method [10] is based on the illuminant invariance feature, which is robust in shadowy and varying illumination conditions. The method is able to fit the road well when the road boundaries are curved (see Figure 23, Rows 1 and 8). However, it relies on the details of road images, so it has relatively high sensitivities to scale and noise compared to Kong *et al*.’s method [9] and our proposed method. In addition, Álvarez *et al*.’s method would be suboptimal when the features of the road and background are too similar (see Figure 23, Rows 3, 4 and 7) or some parts of the road are quite different from other road regions (see Figure 23, Rows 2 and 8).

Our proposed method includes both advantages of Kong *et al*.’s [9] and Álvarez *et al*.’s method [10].The method exploits the road vanishing point for seed selection and high-level information-based refinement, which make it depend less on the details of road images; thus, the method has low sensitivities to scale and noise. Moreover, GrowCut makes it possible to detect road regions when features of the road and background are quite similar. As shown in Figure 23, Rows 4 and 7, the proliferation of seeds will stop near the road boundaries.

## 6. Conclusions and Future Works

This paper presents a hierarchical road detection approach using a vision sensor. The major contributions of this work include: (1) an MPGA-based method proposed for efficient road vanishing point detection; (2) a road vanishing point and clustering-based strategy presented for unsupervised seed selection; (3) a GrowCut-based approach applied at the superpixel level introduced to obtain the initial road segment; and (4) a high level information-based method proposed to refine the road segment by using the CRF framework. Experimental results of the common performance, scale sensitivity and noise sensitivity evaluation validate the effectiveness and robustness of our proposed approach.

Our future work will focus on applying the proposed method to an SoC (system-on-a-chip) for some robot navigation applications, such as map building, path planning, *etc*.
